# No pervasive relationship between phyllosphere nitrifier abundance and canopy nitrification in European forests

**DOI:** 10.1093/nsr/nwag147

**Published:** 2026-03-10

**Authors:** Yong Zhang, Ji Chen, Feng Zhang, Xingwu Duan, Xiaoli Cheng, Jingyun Fang, Peter B Reich

**Affiliations:** State Key Laboratory of Vegetation Structure, Function and Construction (VegLab), School of Ecology and Environmental Science, Yunnan University, China; State Key Laboratory of Loess Science, Institute of Earth Environment, Chinese Academy of Sciences, China; State Key Laboratory of Loess Science, Institute of Earth Environment, Chinese Academy of Sciences, China; CAS Key Laboratory of Tropical Forest Ecology, Xishuangbanna Tropical Botanical Garden, Chinese Academy of Sciences, China; State Key Laboratory of Vegetation Structure, Function and Construction (VegLab), School of Ecology and Environmental Science, Yunnan University, China; State Key Laboratory of Vegetation Structure, Function and Construction (VegLab), School of Ecology and Environmental Science, Yunnan University, China; State Key Laboratory of Vegetation Structure, Function and Construction (VegLab), College of Urban and Environmental Sciences, Peking University, China; Department of Forest Resources, University of Minnesota, USA; Hawkesbury Institute for the Environment, Western Sydney University, Australia; Institute for Global Change Biology and School for Environment and Sustainability, University of Michigan, USA

**Keywords:** functional genes, stable isotopes, canopy nitrification

A recent study inferred the substantial contribution of phyllosphere nitrifiers to canopy nitrification in European forests. By re-analyzing the data from European forests, however, we show no clear relationship between phyllosphere nitrifiers abundance and canopy nitrification. A lack of such relationship may be attributed to uncertainties in canopy nitrification and phyllosphere nitrifiers, calling for a need of interdisciplinary collaboration among empirical, theoretical and modeling scientists.

The discovery of canopy nitrification one century ago provided new insights into the plant–atmosphere exchange of nitrogen [1–3], but the mechanisms driving canopy nitrification remain poorly understood. Recently, Guerrieri *et al.* [4] inferred a substantial contribution of phyllosphere nitrifiers to canopy nitrification in European forests by combining stable isotopes with molecular analyses. This inference was loosely based on the presence of some autotrophic nitrifiers on leaf surfaces in European forests and on assumptions about their rates of nitrification. In fact, a DNA-based method by them cannot distinguish between active and inactive cells for microbes [5]. While we applaud their efforts in managing so many experimental stations and revealing the widespread canopy nitrification, we believe that additional efforts should be taken to provide direct evidence regarding how phyllosphere nitrifiers influence canopy nitrification.

To analyze the relationships between canopy nitrification and the abundance of nitrifiers, we selected observational data by Guerrieri *et al.* [4] in nine European forests (that is, Alice Holt, Bertiz, Brasschaat, Collelongo, Haguenau, Hoeilaart, Rogate, Schänis and Thetford; please note that Hoka was excluded due to the absence of isotopic data). Pearson correlation analysis was used to assess a linear relationship, which was considered significant if *P* < 0.05. When the linear relationship was not significant, we further considered quadratic and multivariate models of regression. Formal analysis and data visualization were performed by using R version 4.2.3.

A re-analysis of raw data shows no clear relationships between canopy nitrification and the abundance of phyllosphere nitrifiers or their components (*P* > 0.05; Fig. 1A, Table S1). Canopy nitrification also has no significant relationships with the ratio of archaeal *amoA* (the gene encoding ammonia monooxygenase subunit A) (AOA) to bacterial *amoA* (AOB), and the ratio of archaeal nitrifiers (AN) to bacterial nitrifiers (BN) in the phyllosphere. We further test their inference by summing nitrifiers in both the phyllosphere (permanent residents) and throughfall (floating residents), as the sum is closer to the actual value of canopy nitrifiers. Again, the relationship between canopy nitrification and total abundance of nitrifiers is non-significant (*P* > 0.05; Fig. 1C). These results weaken the robustness of their inference.

**Figure 1.**
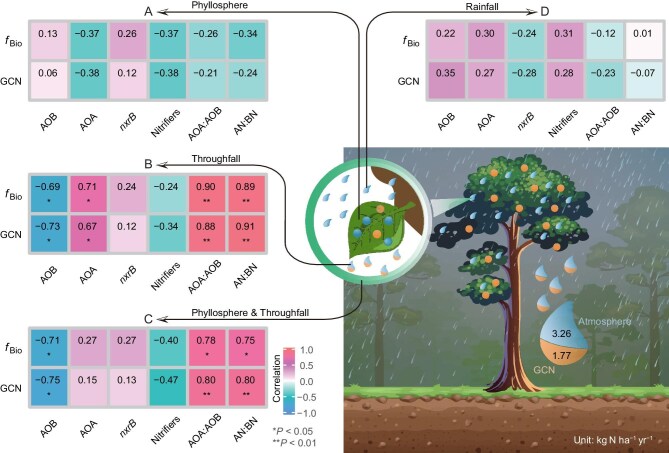
Relationships between tree canopy nitrification and the abundance of nitrifiers in the (A) phyllosphere, (B) throughfall, (C) phyllosphere and throughfall, and (D) rainfall. GCN, gross canopy nitrification; *f*_Bio_, nitrate fraction from GCN; *amoA*, the gene encoding ammonia monooxygenase subunit A; *nxrB*, the gene encoding nitrite oxidoreductase subunit B; AOA, archaeal *amoA*; AOB, bacterial *amoA*; AN, archaeal nitrifiers (that is, AOA); BN, bacterial nitrifiers (that is, AOB and *nxrB*). Gene abundances expressed as log copies per ng DNA (*n* = 9). Tree illustration adapted from Guerrieri *et al.* [4] under a Creative Commons license CC BY 4.0.

We do find some significant relationships in the throughfall (*P* < 0.05; Fig. 1B). Specifically, canopy nitrification decreases with throughfall AOB abundance, while it increases with throughfall AOA abundance, AOA:AOB and AN:BN. This may imply that floating AOA derived from the canopy plays a dominant role in canopy nitrification [6]. However, total AOA abundance in the canopy (phyllosphere and throughfall) is not related to canopy nitrification (*P* > 0.05; Fig. 1C). At the same time, a non-significant relationship between canopy nitrification and rainfall AOA abundance (*P* > 0.05; Fig. 1D, Table S2) further suggests that rainfall is not a main source of throughfall AOA, and hence the potential of dry deposition-derived AOA cannot be excluded [7]. All the aforementioned results reflect potential uncertainties in identifying the contribution of phyllosphere nitrifiers to canopy nitrification.

What is causing these uncertainties? First, microbial nitrification in the throughfall is not equivalent to canopy nitrification. When rainfall contacts plants, it is partitioned into interception, throughfall and stemflow. Although the fraction of interception can be negligible, a large amount of stemflow comes from the leaf surface by passing through petioles and branches [8]. For example, leaves of *Fagus sylvatica* and *Pinus sylvestris* (the species investigated by Guerrieri *et al.* [4]) tightly surround their stems (Fig. 2A and B). If leaf-derived stemflow (LS) is considered, canopy nitrification (*F*_Bio,canopy_) can be revised as:


(1) \begin{eqnarray*}
&&{F}_{{\mathrm{Bio, canopy}}} = \textit{Canopy}\ \textit{nitrate}\ \textit{flux}\\
&&\times \frac{{{f}_{{\mathrm{Bio, throughfall}}}\times {F}_{{\mathrm{Bio, throughfall}}}
+ {f}_{{\mathrm{Bio, LS}}}\times {F}_{{\mathrm{Bio, LS}}}}}{{{F}_{{\mathrm{Bio, throughfall}}}\times {F}_{{\mathrm{Bio, LS}}}}},\\
\end{eqnarray*}

**Figure 2.**
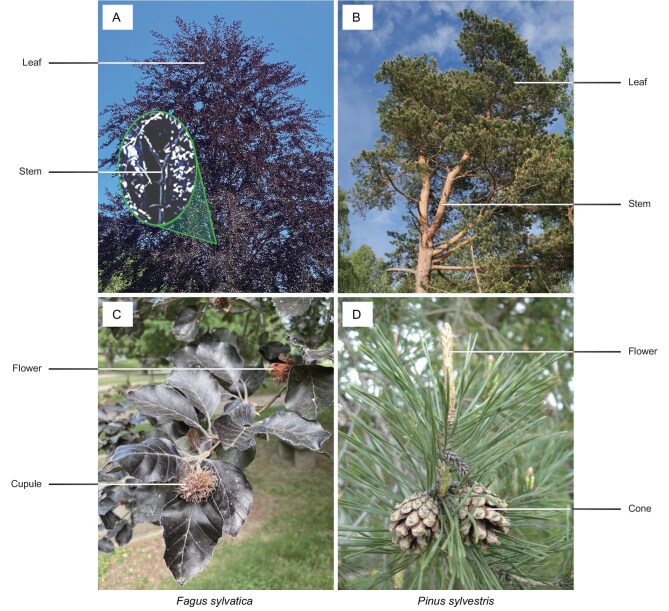
Canopy morphology of *F. sylvatica* (A and C) and *P. sylvestris* (B and D). (A) and (C) Adapted from Awinch1001 at Wikipedia under a Creative Commons license CC BY-SA 4.0. (B) Adapted from Mickaël Delcey at Wikipedia under a Creative Commons license CC BY-SA 3.0. (D) Adapted from Georgi Kunev at Wikipedia under a Creative Commons license CC BY 2.5.

where *F*_Bio_ is biological nitrate flux, and *f*_Bio_ is nitrate fraction from *F*_Bio_.

Second, a full view of phyllosphere nitrifiers is lacking. Guerrieri *et al.* [4] only considered a part of nitrifiers on leaf surfaces, that is AOB, AOA and *nxrB* (the gene encoding nitrite oxidoreductase subunit B). However, some studies document the presence of other nitrifying genes in both the phyllosphere [9] and precipitation [8], such as *nxrA* (the gene encoding nitrite oxidoreductase subunit A) and comammox *amoA*. Fungal nitrifiers capable of heterotrophic nitrification have recently received more attention as well [10]. In addition, the phyllosphere comprises the aerial parts of plants and is dominated by the leaves, flowers and fruits [11]. Although all these parts exist for *F. sylvatica* and *P. sylvestris* (Fig. 2C and D), the authors only collected leaves.

Third, there still are some inherent limitations for both stable isotopes and molecular analyses. Isotopic signatures are hypervariable, due to unequal ecosystem coverage, over-reliance of key parameters on literature, lack of canonical models, and untested assumptions [12,13]. For example, so far there is no atmospheric fractionation line of oxygen isotopes [14], and studies often adopt the terrestrial fractionation line (a coefficient of 0.52) when analyzing atmospheric oxygen isotopes [2,4,15]. On the other hand, quantitative polymerase chain reaction (qPCR) approaches and 16S rRNA sequencing only provide limited information of nitrifiers [5]. For example, qPCR approaches are often limited by the availability and efficiency of PCR primers [5], as well as the capacity of simultaneous PCR reactions [16].

Nevertheless, it is exciting to see Guerrieri and colleagues’ attempt to unlock the mechanisms driving canopy nitrification in European forests. As one of the pioneers in canopy nitrification, Guerrieri has done some excellent work [2,4,15]. A non-significant relationship between phyllosphere nitrifier abundance and canopy nitrification in European forests (Fig. 1) is surprising since, in theory, nitrifiers are responsible for nitrification. Although some inherent limitations cannot shift overnight, we believe that uncertainties in canopy nitrification and phyllosphere nitrifiers will diminish as empirical, theoretical and modeling scientists intensify efforts. It will then be possible to clarify how phyllosphere nitrifiers influence canopy nitrification.

## Supplementary Material

nwag147_Supplemental_File

## Data Availability

All data are available from Zenodo (https://doi.org/10.5281/zenodo.10302622).
