# Supplementary material for: No pervasive relationship between phyllosphere nitrifier abundance and canopy nitrification in European forests
Source: Natl Sci Rev. 2026 Mar 10;13(9):nwag147. doi: 10.1093/nsr/nwag147 (PMC13220754; doi:10.1093/nsr/nwag147)
Supplement: nwag147_Supplemental_File [file nwag147_supplemental_file.docx]

**Supplementary Information**

Yong Zhang^1,2^, Ji Chen ^2^, Feng Zhang^3^, Xingwu Duan ^1*^, Xiaoli Cheng ^1*^, Jingyun Fang ^4^, Peter B. Reich^5,6,7^

^1^State Key Laboratory of Vegetation Structure, Function and Construction (VegLab), School of Ecology and Environmental Science, Yunnan University, Kunming, China

^2^State Key Laboratory of Loess Science, Institute of Earth Environment, Chinese Academy of Sciences, Xi'an, China

^3^CAS Key Laboratory of Tropical Forest Ecology, Xishuangbanna Tropical Botanical Garden, Chinese Academy of Sciences, Mengla, China

^4^State Key Laboratory of Vegetation Structure, Function and Construction (VegLab), College of Urban and Environmental Sciences, Peking University, Beijing, China

^5^Department of Forest Resources, University of Minnesota, St. Paul, MN, USA

^6^Hawkesbury Institute for the Environment, Western Sydney University, Penrith, New South Wales, Australia

^7^Institute for Global Change Biology and School for Environment and Sustainability, University of Michigan, Ann Arbor, MI, USA

^*^Corresponding author: X.D. ([xwduan@ynu.edu.cn](mailto:xwduan@ynu.edu.cn)) or X.C. ([xlcheng@ynu.edu.cn](mailto:xlcheng@ynu.edu.cn)).

**Table S1 |** Regression analysis between tree canopy nitrification and the abundance of nitrifiers in the phyllosphere. Here we considered quadratic and multivariate models of regression. GCN, gross canopy nitrification; *f*_Bio_, nitrate fraction from GCN; *amoA*, the gene encoding ammonia monooxygenase subunit A; *nxrB*, the gene encoding nitrite oxidoreductase subunit B; AOA, archaeal *amoA*; AOB, bacterial *amoA*; AN, archaeal nitrifiers (i.e., AOA); BN, bacterial nitrifiers (i.e., AOB & *nxrB*). Gene abundances expressed as log copies per ng DNA (*n* = 9).

| Model | *F* | *R^2^* | *p* |
| --- | --- | --- | --- |
| *f*_Bio_ ~ poly(AOB, 2) | 0.323 | 0.097 | 0.736 |
| *f*_Bio_ ~ poly(AOA, 2) | 0.699 | 0.189 | 0.534 |
| *f*_Bio_ ~ poly(*nxrB*, 2) | 0.852 | 0.221 | 0.473 |
| *f*_Bio_ ~ poly(Nitrifiers, 2) | 0.706 | 0.191 | 0.530 |
| *f*_Bio_ ~ poly(AOA:AOB, 2) | 0.302 | 0.092 | 0.750 |
| *f*_Bio_ ~ poly(AN:BN, 2) | 0.472 | 0.136 | 0.645 |
| *f*_Bio_ ~ AOB + AOA + *nxrB* | 1.295 | 0.437 | 0.373 |
| GCN ~ poly(AOB, 2) | 0.337 | 0.101 | 0.727 |
| GCN ~ poly(AOA, 2) | 0.516 | 0.147 | 0.621 |
| GCN ~ poly(*nxrB*, 2) | 0.754 | 0.201 | 0.510 |
| GCN ~ poly(Nitrifiers, 2) | 0.522 | 0.148 | 0.618 |
| GCN ~ poly(AOA:AOB, 2) | 0.307 | 0.093 | 0.747 |
| GCN ~ poly(AN:BN, 2) | 0.278 | 0.085 | 0.767 |
| GCN ~ AOB + AOA + *nxrB* | 0.671 | 0.287 | 0.606 |

**Table S2** | Regression analysis between tree canopy nitrification and the abundance of nitrifiers in the rainfall. Here we considered quadratic and multivariate models of regression. GCN, gross canopy nitrification; *f*_Bio_, nitrate fraction from GCN; *amoA*, the gene encoding ammonia monooxygenase subunit A; *nxrB*, the gene encoding nitrite oxidoreductase subunit B; AOA, archaeal *amoA*; AOB, bacterial *amoA*; AN, archaeal nitrifiers (i.e., AOA); BN, bacterial nitrifiers (i.e., AOB & *nxrB*). Gene abundances expressed as log copies per ng DNA (*n* = 9).

| Model | *F* | *R^2^* | *p* |
| --- | --- | --- | --- |
| *f*_Bio_ ~ poly(AOB, 2) | 0.210 | 0.065 | 0.817 |
| *f*_Bio_ ~ poly(AOA, 2) | 0.300 | 0.091 | 0.752 |
| *f*_Bio_ ~ poly(*nxrB*, 2) | 0.389 | 0.115 | 0.694 |
| *f*_Bio_ ~ poly(Nitrifiers, 2) | 0.324 | 0.097 | 0.735 |
| *f*_Bio_ ~ poly(AOA:AOB, 2) | 0.095 | 0.031 | 0.910 |
| *f*_Bio_ ~ poly(AN:BN, 2) | 0.195 | 0.061 | 0.828 |
| *f*_Bio_ ~ AOB + AOA + *nxrB* | 0.415 | 0.199 | 0.750 |
| GCN ~ poly(AOB, 2) | 0.438 | 0.127 | 0.665 |
| GCN ~ poly(AOA, 2) | 0.310 | 0.094 | 0.744 |
| GCN ~ poly(*nxrB*, 2) | 0.574 | 0.161 | 0.592 |
| GCN ~ poly(Nitrifiers, 2) | 0.335 | 0.100 | 0.728 |
| GCN ~ poly(AOA:AOB, 2) | 0.182 | 0.057 | 0.838 |
| GCN ~ poly(AN:BN, 2) | 0.162 | 0.051 | 0.854 |
| GCN ~ AOB + AOA + *nxrB* | 0.648 | 0.280 | 0.617 |
